# Reactive oxygen and nitrogen species induce protein and DNA modifications driving arthrofibrosis following total knee arthroplasty

**DOI:** 10.1186/1755-1536-2-5

**Published:** 2009-11-13

**Authors:** Theresa A Freeman, Javad Parvizi, Craig J Della Valle, Marla J Steinbeck

**Affiliations:** 1 Department of Orthopaedic Surgery, Thomas Jefferson University, 1015 Walnut Street, Suite 501, Philadelphia, PA 19107, USA; 2 The Rothman Institute of Orthopedics at Thomas Jefferson University, Market Street Philadelphia, PA 19107, USA; 3 Department of Orthopaedic Surgery, Rush University Medical Center, 1725 W Harrison Street, Suite 1063, Chicago, IL 60612, USA; 4 Department of Biomedical Engineering and Department of Drexel Medicine, Drexel University, 3120 Market Street, 323 Bossone, Philadelphia, PA 19104, USA

## Abstract

**Background:**

Arthrofibrosis, occurring in 3%-4% of patients following total knee arthroplasty (TKA), is a challenging condition for which there is no defined cause. The hypothesis for this study was that disregulated production of reactive oxygen species (ROS) and nitrogen species (RNS) mediates matrix protein and DNA modifications, which result in excessive fibroblastic proliferation.

**Results:**

We found increased numbers of macrophages and lymphocytes, along with elevated amounts of myeloperoxidase (MPO) in arthrofibrotic tissues when compared to control tissues. MPO expression, an enzyme that generates ROS/RNS, is usually limited to neutrophils and some macrophages, but was found by immunohistochemistry to be expressed in both macrophages and fibroblasts in arthrofibrotic tissue. As direct measurement of ROS/RNS is not feasible, products including DNA hydroxylation (8-OHdG), and protein nitrosylation (nitrotyrosine) were measured by immunohistochemistry. Quantification of the staining showed that 8-OHdg was significantly increased in arthrofibrotic tissue. There was also a direct correlation between the intensity of inflammation and ROS/RNS to the amount of heterotopic ossification (HO). In order to investigate the aberrant expression of MPO, a real-time oxidative stress polymerase chain reaction array was performed on fibroblasts isolated from arthrofibrotic and control tissues. The results of this array confirmed the upregulation of MPO expression in arthrofibrotic fibroblasts and highlighted the downregulated expression of the antioxidants, superoxide dismutase1 and microsomal glutathione S-transferase 3, as well as the significant increase in thioredoxin reductase, a known promoter of cell proliferation, and polynucleotide kinase 3'-phosphatase, a key enzyme in the base excision repair pathway for oxidative DNA damage.

**Conclusion:**

Based on our current findings, we suggest that ROS/RNS initiate and sustain the arthrofibrotic response driving aggressive fibroblast proliferation and subsequent HO.

## Background

A number of factors are known to result in complications after total knee arthroplasty (TKA), which include preoperative deformity, neuromuscular disease, patient noncompliance with rehabilitation protocol and technical errors such as component malpositioning [1-4]. A separate portion of the patient population develops arthrofibrosis after TKA, clinically defined as abnormal scarring of the joint in which the formation of dense fibrous tissue and tissue metaplasia prevent normal range of motion [4-11]. For these patients, surgical intervention and revision arthroplasty leads to a worsening of the fibrotic condition and eventual disability [4,9,10].

The exact pathoaetiology of arthrofibrosis following TKA remains elusive. However, aggressive fibroblast proliferation and tissue metaplasia are a hallmark of this condition [10,12]. Our previous studies highlighted multiple tissue changes including the presence of pro-inflammatory factors, increased cell proliferation, survival and increased matrix deposition [11,13]. In addition, we showed that mast cells, hypoxia and hypoxia-associated oxidative stress are linked to the progression of the metaplastic changes, fibrocartilage formation and heterotopic ossification observed in idiopathic arthrofibrosis [13].

Normally, tissue repair occurs through a sequence of coordinated events that lead to the eventual restoration of tissue form and function. The healing response is initiated by the clotting cascade, which results in the migration of inflammatory cells (neutrophils and monocytes) to the site of injury [14]. Inflammatory cell infiltration is followed by the recruitment of fibrocytes that undergo proliferation, differentiation and the ultimate deposition of an organized matrix [15-17]. Both the migration of inflammatory cells into the injured tissue and the proliferation of fibroblasts results in the release of cytokines, growth factors and reactive oxygen and nitrogen species (ROS/RNS) [18-26]. Thus, an intricate balance between cell proliferation, matrix production and tissue remodelling is in place during normal healing, and the restoration of tissue integrity is dependent on the coordinated or 'coupled' function of inflammatory cells responsible for remodelling, and fibroblasts, the cells responsible for resynthesis of the matrix. Once the process of healing nears completion, the majority of the inflammatory cells undergo apoptosis, the tissue heals and the release of ROS/RNS and other factors, which are no longer necessary, halts. Therefore, resolution of the inflammatory response is critical to the restoration of the tissue to a functional state and the prevention of fibrosis [16,17].

Disruption of the ROS/RNS equilibrium, caused by overproduction or inefficient antioxidant response, has been implicated in the pathoaetiology of fibrotic conditions including retroperitoneal fibrosis [27], Dupuytren's [28-31], scleroderma [32,33] and Crohn's disease [34]. In addition, chronic inflammation and oxidative stress contribute to genomic DNA damage. In Crohn's disease, this damage leads to the overexpression of p53, which potentially contributes to the loss of cell cycle control [35].

Our hypothesis was that susceptible patients exhibit excessive production and/or the inefficient removal of ROS/RNS after undergoing TKA surgery, which leads to aggressive fibrosis. Therefore, we performed a series of interlinked molecular studies to evaluate ROS/RNS modifications, inflammation and ROS/RNS responsive gene expression as part of the pathogenesis of arthrofibrosis following TKA.

## Results

### Patient cohort information

The clinical records of patients were reviewed in detail to extract variables including age, sex, body mass index (BMI), years post initial surgery, pre-existent co-morbidities, functional scores (particularly the details of range of motion) and all other relevant information (Table 1). Ten patients undergoing uneventful primary TKA were matched for age, sex and BMI and included as the control cohort.

**Table 1:** Patient information for the arthrofibrotic cohort.

Sex	Males	3
	Females	7

**Average age**		

	Males	63.7 ± 12.5

	Females	57.6 ± 3.7

**Years post initial total knee arthroplasty**		

	Males	3.0 ± 1.9

	Females	2.9 ± 0.9

**Average body mass index (kg/cm^2^)**		

	Males	28.4 ± 1.6

	Females	36.4 ± 1.8

**Range of motion (0°-100° flexion)**		

	Males	53.3 ± 10.8

	Females	44.2 ± 6.6

**Tissue calcification (bone volume, mm^3 ^of hydroxyapatite)**		

	Males	2.04 ± 2.34

	Females	3.93 ± 2.47

There was no correlation between most of the demographic factors, range of motion and the molecular findings in this cohort of patients. The only significant correlation observed was between range of motion (ROM) and tissue calcification/bone volume (BV) (Table 1) [13]. As no other correlations were evident, all subsequent analyses were grouped based on patient tissue BV. The control tissue BV was 0.005 ± 0.01.

### Monocyte and lymphocyte infiltration

**Figure 1:**
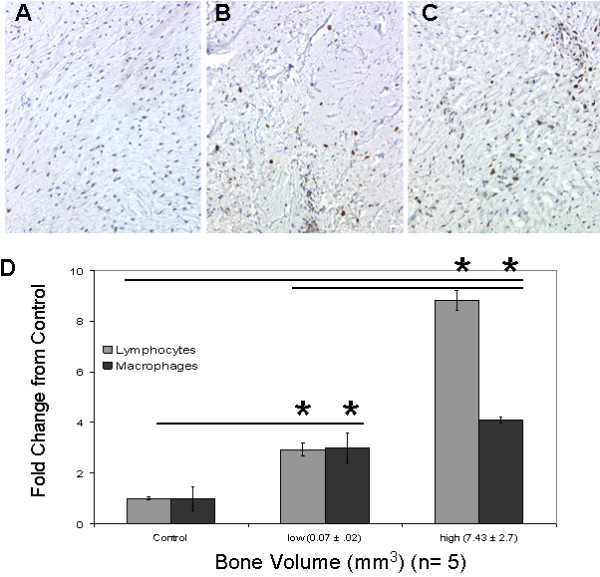
**Patients were divided into to three cohorts; control before revision, and based on the amount of tissue calcification detected by microcomputed tomography, a low bone volume (BV) group and a high BV group**. A - C show representative images of CD68 immunohistochemistry on (A) control tissue and (B) low BV and (C) high BV arthrofibrotic tissue. Note the large increase in the presence of macrophages in the fibrotic regions of the high BV arthrofibrotic periarticular tissue. Image analysis of lymphocytes (Giemsa stain) and macrophage was based on percentage positive per total cell number for each patient cohort. (D) There was a correlative increase in lymphocyte and macrophage numbers with tissue calcification. The fold increases for both BV groups were statistically significant as compared to control and each other (*; *P *< 0.05). Magnification 100× insets, micron bar equals 100 μm.

**Figure 2:**
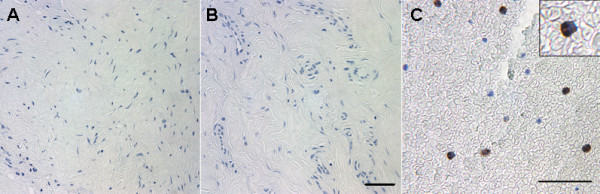
**Immunohistochemical stain for neutrophil elastase of the periarticular tissue from patients with arthrofibrosis**. Representative immunohistochemical results are shown for (A) elastase in the fibrous and (B) in the vascular regions of the tissue. A positive control of a neutrophil rich bone marrow sample is shown in (C). Magnification 200× with 400× insets, micron bar equals 100 μm.

Histological analysis of the 10 control tissues showed ~1% average inflammatory cell presence (Figure 1A). This is in contrast to the arthrofibrotic tissues, which showed a 3.0 - 4.0-fold increase in the number of macrophages and a 3.0 - 9-fold increase in lymphocytes as compared to control (Figure 1B, C, D). The inflammatory response did not include the infiltration of neutrophils (Figure 2). The absence of these cells suggests that the inflammation was not due to infection. Image analysis of the fibrotic regions (non-calcified areas) showed increased inflammation in patient tissues in both the low and high BV groups (Figure 1D). The increase in macrophage and lymphocyte numbers were significant in the both BV groups relative to control tissue (*P *< 0.05).

### Expression of myeloperoxidase (MPO) in arthrofibrotic tissue

**Figure 3:**
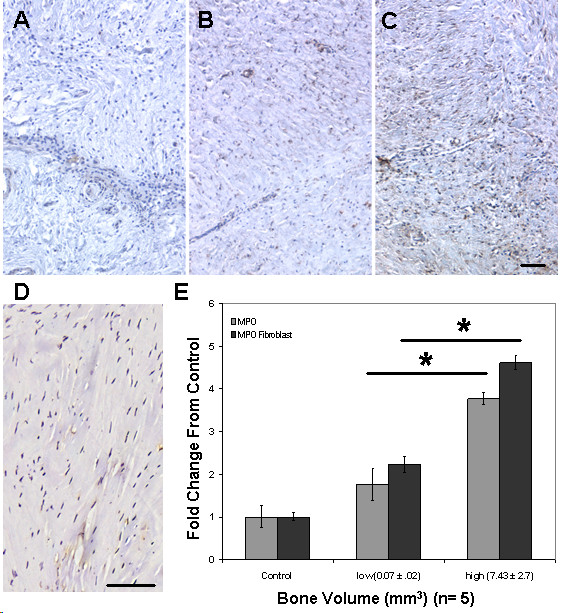
**Myeloperoxidase (MPO) immunohistochemistry and image analysis showing increased expression of MPO in macrophages and fibroblasts within the fibrotic regions of the arthrofibrotic tissue**. A - C show representative images for (A) control tissue and (B) low bone volume (BV) and (C) high BV arthrofibrotic tissue. Note the large increase in the presence of MPO positive macrophages (round cells) and fibroblasts (elongated cells) in the fibrotic regions of the 'high' bone arthrofibrotic periarticular tissue. (D) A representative image of MPO expression in tissue from the non-arthrofibrotic stiff knee cohort, showing that MPO was not expressed by control tissue fibroblasts. Image analysis of the patient cohorts was based on the average percentage positive per total cell number. There was a correlative increase in MPO expression with tissue calcification. The fold increases for the high BV group were statistically significant as compared to control and to the low BV group (*; *P *< 0.05). Magnification 100× for (A -C), micron bar equals 100 μm.

Based on the increased presence of macrophages in the arthrofibrotic tissues, subsequent analyses to determine the levels of MPO were performed. MPO is an enzyme that produces highly reactive products that mediate chlorination, protein/DNA hydroxylation and protein/DNA nitrosylation [36-41]. Low levels of MPO were detected in control tissue macrophages by immunohistochemistry. A representative tissue image is shown in Figure 3A. Figures 3B and 3C show the elevated expression of this enzyme in macrophages within the arthrofibrotic tissue. Although less intense, MPO expression was also observed in the periarticular fibroblasts, which was not observed in control tissues (Figure 3D). Image analysis revealed a correlative increase in macrophage (based on CD68 image analysis) and fibroblast expression of MPO in arthrofibrotic patient tissues (Figure 3E). This increase was statistically significant for the high BV group compared to the control group (*P *< 0.05) and the low BV group approached significance.

### Fibrotic tissues contain byproducts of reactive oxygen and nitrogen species

**Figure 4:**
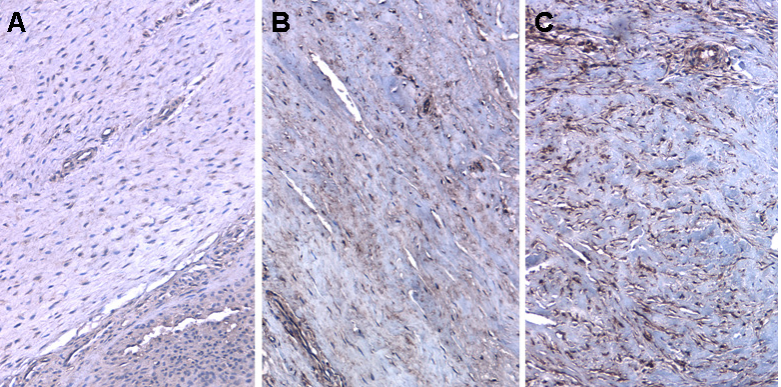
**Immunohistochemical analysis of reactive oxygen species/reactive nitrogen species byproducts in periarticular tissue from patients diagnosed with arthrofibrosis**. A-C show representative nitrotyrosine immunohistochemistry on (A) control tissue and (B) low bone volume (BV) and (C) high BV arthrofibrotic tissue. Note the large increase in the presence of nitrotyrosine in the fibrotic regions associated with tissues containing high amounts of bone. Magnification 100× for (A-C), micron bar equals 100 μm.

**Figure 5:**
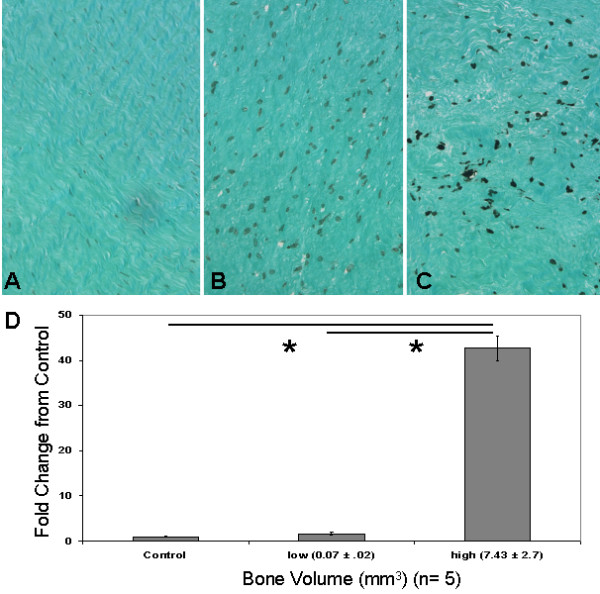
**Immunohistochemical and image analysis of reactive oxygen species (ROS)/reactive nitrogen species (RNS) byproducts in periarticular tissue from patients diagnosed with arthrofibrosis**. A-C show representative images of 8-OHdG immunohistochemistry on (A) control tissue and (B) low bone volume (BV) and (C) high BV arthrofibrotic tissue. Note the large increase in the presence of 8-OHdG in the fibrotic regions associated with tissues containing high amounts of bone. Image analysis of the patient cohorts was based on the average percentage positive per total cell number. (D) The fold increases in ROS/RNS product formation for the high BV group were statistically significant as compared to control and the low BV group (*; *P *< 0.05). Magnification 100× for (A-C), micron bar equals 100 μm.

Based on the increased MPO in the arthrofibrotic tissues, subsequent analysis were performed in order to determine the levels of ROS/RNS. As direct measurement of ROS/RNS production in tissues lacks sensitivity and reproducibility, we measured protein nitrosylation and DNA hydroxylation (8-OHdG), both are end products of ROS/RNS-mediated reactions. There was no detectable protein nitrosylation (Figure 4A) or 8-OHdG modifications (Figure 5A) in control tissues by immunohistochemistry. Evident within the fibrotic regions of the arthrofibrotic tissues were nitrosylated proteins (Figure 4B and 4C) and cells containing 8-OHdG (Figure. 5B and 5C). The ROS/RNS-mediated 8-OHdG nuclear modification was localized to regions of high fibroblast density and was significantly increased in tissues in the high BV group (Figure 5D; *P *< 0.05).

### MPO expression and oxidative stress responses of isolated arthrofibrotic fibroblasts

In order to determine MPO expression specific to the arthrofibrotic fibroblast population and to analyse oxidative stress, we performed a real-time polymerase chain reaction (PCR) microarray panel for oxidative stress responsive genes (Table 2). In agreement with the immunohistochemical finding, MPO expression levels in the isolated arthrofibrotic fibroblasts were 2.4-fold higher than control fibroblasts. In contrast to MPO, the expression of the major anti-oxidant enzyme superoxide dismutase 1 (SOD1) was down-regulated 2.9-fold. A number of other oxidative stress responsive genes were also disregulated, showing a significantly increased or decreased expression by arthrofibrotic fibroblasts. Of note was a 7.6-fold increase in polynucleotide kinase 3'-phosphatase (PNKP), an enzyme involved in the base excision repair pathway for nearly all ROS/RNS-induced DNA mutations, and an 11.9-fold increase in thioredoxin reductase 1 (TrxR1) expression, an oxidoreductase enzyme that promotes cell proliferation. Finally, there was 21.1-fold decrease in the expression of another important anti-oxidant, microsomal glutathione S-transferase 3 (MGST3). SOD1 is a both a cytosolic and a secreted protein, and MGST3 is a major intracellular antioxidant enzyme.

**Table 2:** Oxidative stress and antioxidant defense polymerase chain reaction array

Protein	Δ Expr		Gene
MGST3	-21.1	Microsomal glutathione S-transferase 3	GST-III

PTGS2	-8	Prostaglandin-endoperoxide synthase 2 (prostaglandin G/H synthase and cyclooxygenase)	COX-2/COX2

TPO	-8	Thyroid peroxidase	MSA/TPX

GPX5	-6.06	Glutathione peroxidase 5 (epididymal androgen-related protein)	GPX5

DHCR24	-5.79	24-dehydrocholesterol reductase	Nbla03646/SELADIN1

GPX6	-5.28	Glutathione peroxidase 6 (olfactory)	Gpx6

AOX1	-5.04	Aldehyde oxidase 1	AO/AOH1

STK25	-4.59	Serine/threonine kinase 25 (STE20 homolog, yeast)	DKFZp686J1430/SOK1

NOX5	-4.59	NADPH oxidase, EF-hand calcium binding domain 5	NOX5A/NOX5B

FOXM1	-3.91	Forkhead box M1	FKHL16/FOXM1B

TXNRD2	-3.65	Thioredoxin reductase 2	SELZ/TR

CYGB	-3.4	Cytoglobin	HGB/STAP

OXR1	-3.32	Oxidation resistance 1	Nbla00307

SOD1	-2.89	Superoxide dismutase 1, soluble (amyotrophic lateral sclerosis 1 (adult))	ALS/ALS1

MPV17	-2.89	MpV17 mitochondrial inner membrane protein	SYM1

GPR156	-2.83	G protein-coupled receptor 156	GABABL/PGR28

DUOX2	-2.52	Dual oxidase 2	LNOX2/NOXEF2

PTGS1	-2.52	Prostaglandin-endoperoxide synthase 1 (prostaglandin G/H synthase and cyclooxygenase)	COX1/COX3

PRDX2	-2.35	Peroxiredoxin 2	NKEFB/PRP

MPO	2.351	Myeloperoxidase	myeloperoxidase

GPX1	2.462	Glutathione peroxidase 1	GSHPX1

NOS2A	2.764	Nitric oxide synthase 2A (inducible, hepatocytes)	HEP-NOS/INOS

SIRT2	3.102	Sirtuin (silent mating type information regulation 2 homolog) 2 (S. cerevisiae)	SIR2L/SIR2L2

NME5	3.403	Non-metastatic cells 5, protein expressed in (nucleoside-diphosphate kinase)	NM23-H5/NM23H5

PRNP	4.387	Prion protein (p27-30) (Creutzfeldt-Jakob disease, Gerstmann-Strausler-Scheinker syndrome, fatal familial insomnia)	ASCR/CD230

SGK2	5.924	Serum/glucocorticoid regulated kinase 2	H-SGK2

LPO	6.277	Lactoperoxidase	SPO

PNKP	7.639	Polynucleotide kinase 3'-phosphatase	PNK

TXNRD1	11.85	Thioredoxin reductase 1	GRIM-12/TR

GPX3	13.3	Glutathione peroxidase 3 (plasma)	GPx-P/GSHPx-3

## Discussion

This study highlights several major findings that are relevant to the pathogenesis of arthrofibrosis. First, histological findings show an increase in the number of macrophages and lymphocytes in the periarticular tissue of patients with arthrofibrosis. Second, by demonstrating the atypical presence of MPO and ROS/RNS products (oxidized DNA and nitrosylated proteins) in arthrofibrotic tissues, a potentially important mechanism involved in gene disregulation has been identified. Finally, by immunohistochemistry and microarray analysis, we show aberrant expression of MPO, SOD1 and other oxidative stress genes including; TrxR1, PNKP and MGST3, which all show significant fold changes in arthrofibrotic fibroblasts. Disregulated gene expression of the cellular oxidant/anti-oxidant system in fibroblasts implicates their involvement in the abnormal fibroblast proliferation, survival and hypertrophic formation of scar tissue.

No in depth studies have been performed on arthrofibrotic tissue after TKA in order to determine the extent of inflammation or other molecular mechanisms involved in this disease process [10,11,13]. However, chronic inflammation has been associated with the development of fibrosis in other tissues, such as Dupytren's Contracture [28,30,31,42,43], Crohn's disease [34] and additional tissues [14,44]. Studies have also linked the development of arthrofibrosis after anterior cruciate ligament surgery to the presence of prior inflammation [45] and to an increased infiltration of T cells within 10 days after post surgery [46]. Similarly, in intestinal, pulmonary and renal fibrosis increased numbers of macrophages and mast cells are found within the granulation tissue during the proliferative stage of wound healing [20,26,27,47]. Our findings, that macrophages and lymphocytes are present in the arthrofibrotic tissue, supports the involvement of chronic inflammation in the fibrotic process which develops after TKA. The persistence of this local inflammation, regardless of the number of years post initial surgery, was tightly linked to increased tissue calcification and decreased ROM. This association suggests that calcification and chronic inflammation are connected, which has been previously observed [48-52].

ROS/RNS is a collective term for a growing number of reactive species [37,53,54]. They are produced by a variety of pro-oxidant enzymes including MPO, which directly produces the highly reactive products hypochlorous acid (HOCl) and chlorine gas (Cl_2_) [36,55]. These products react with other ROS/RNS, leading to the generation of even a greater number of ROS/RNS [36-41,53,54]. In addition, HOCl and Cl_2 _have been shown to mediate modifications of extracellular [56,57] and intracellular components [40,41]. Specifically, collagen is oxidized by a reaction with HOCl and Cl_2_, resulting in the formation of chlorinated products. These modifications can affect the organization of the tissue matrix, altering its mechanical properties as well as preventing normal remodelling and resolution of the injury response. Intracellular modifications by MPO include ROS/RNS-mediated DNA hydroxylation and other base pair modifications, which affect gene expression.

In the present study, we show periarticular arthrofibrotic tissue with macrophages and fibroblasts uncharacteristically expressing high levels of MPO. The over-expression of MPO by macrophages and non-myeloid cells is also an aetiology associated with cystic fibrosis and hepatic fibrosis [58,59]. The promiscuous expression in these fibrotic diseases is associated with a -463G/A polymorphism within the MPO promoter [58,59]. Individuals can be born with this polymorphism or develop it as a result of ROS/RNS-mediated promoter mutagenesis [36-41,58,59]. Our findings suggest that MPO expression by fibroblasts may have a related aetiology and that MPO may be a driving force in the fibrotic process associated with arthrofibrosis.

ROS/RNS play diverse roles in wound healing - they regulate mast cell degranulation and the release of a number of enzymes, cytokines and growth factors (for example, transforming growth factor-β and connective tissue growth factor (CTGF)) that participate in normal wound healing and fibrosis [23]. They can also directly stimulate CTGF expression, fibroblast proliferation and matrix production [33,37,60]. In normal wound healing, the majority of inflammatory cells undergo apoptosis, the tissue heals and the release of ROS/RNS and other factors stop, thereby ending the cycle of proliferation and allowing for wound resolution [61]. In the arthrofibrotic tissue, however, we see the presence of inflammatory cells and ROS/RNS products years after the initial TKA.

We also observed a decreased expression of SOD1, which can exacerbate oxidative stress. An imbalanced and inefficient antioxidant response has been noted in other chronic inflammatory conditions. If the antioxidant response is not sufficient, as in Crohn's disease where an overall increase in ROS/RNS results because of an excessive accumulation of MPO in the tissue coupled with a decrease in hydroxyl radical scavengers, the balance is shifted [34]. As a result of the disturbed ROS/RNS equilibrium, the inflammatory and proliferative phases of wound healing do not resolve and an aggressive fibrotic response ensues.

Since ROS/RNS mediate downstream effects, a second confounding factor(s) must be involved in disease progression. The other important observation in our study was the increased hydroxylation of DNA, signifying DNA modification. Additional supportive evidence for DNA oxidative damage-induced modifications in arthrofibrotic fibroblasts was the 7.6-fold upregulation of PNKP, a key enzyme in the base excision repair pathway [62]. Specifically, PNKP is one of the primary proteins responsible for repair of oxidatively-induced DNA lesions and single strand breaks. In addition to the upregulation of PNKP, the expression of the oxidative stress responsive gene TrxR1 was increased 11.9-fold in arthrofibrotic fibroblasts. TrxR1 is an oxidoreductase enzyme that promotes cell growth, down-regulates the function of p53 induced apoptosis, regulates DNA synthesis and protects against oxidant damage [63]. Finally, there was 21.1-fold decrease in the expression of MGST3, an anti-oxidant enzyme that reduces lipid hydroperoxides and detoxifies lipid peroxidation end products such as 4-hydroxynonenal [64].

## Conclusion

There was an increase in inflammatory cell numbers, MPO expression, ROS/RNS product accumulation and disregulation of ROS/RNS responsive gene expression. The overexpression of MPO and the presence of ROS/RNS products indicates that ROS/RNS production by inflammatory cells and fibroblasts within the arthrofibrotic tissue is excessive, due either to increased MPO or to the decreased cellular antioxidant expression of SOD1 and MGST3 or a combination of both. These observations strongly suggest an imbalance in the oxidant/anti-oxidant system regulating the normal resolution of the inflammatory and fibroblastic proliferative phases of wound healing. Based on these findings, we believe that aggressive periarticular fibrosis and unresolved healing in patients with arthrofibrosis results from an excessive accumulation of ROS/RNS, ROS/RNS-modified DNA and disregulation of oxidative stress responsive genes.

## Methods

### Tissue collection and processing

This multi-centre study used a standardized tissue retrieval protocol allowing collection and analysis of periarticular tissues from the knee of patients undergoing revision arthroplasty for arthrofibrosis. The diagnosis of arthrofibrosis is based on clinical, radiological examination and intra-operative findings [11]. For these patients, the distinct intra-operative findings are extensive fibrotic tissue formation that fills the lateral, medial, and parapatellar gutters, generally within 1 year after TKA. In this study, tissue samples from 10 affected knees and 10 knees from osteoarthritis (OA) patients undergoing primary TKA (controls) were retrieved. Primary surgical tissues controls were chosen for comparison with arthrofibrotic tissues, as these tissues represent the pre-surgical status, where normal OA associated inflammation exists but is not associated with excessive fibrosis or metaplastic changes [65].

Tissue samples were taken from the periarticular area, which included the suprapatellar, medial gutter, lateral gutter and infrapatellar regions. The tissue was wrapped in sterile saline soaked gauze and transferred, or shipped overnight on ice, to the laboratory for fixation and detailed analyses. Tissue from each anatomical location was cut into 2 × 5 mm pieces and, depending on the amount of available tissue, four to five pieces of tissue from one region were placed in a paraffin block. An equal number and distribution of tissue cubes was used for fibroblast isolation. Any remaining tissue was flash frozen in liquid nitrogen and stored at -80°C. Tissue collection was performed in accordance with the Institutional Review Board guidelines of the participating institutes.

### Microcomputed tomography (μCT) analysis

Each of the paraffin blocks containing tissue were subjected to μCT analysis in order to determine heterotopic ossification (Scanco μCT 40, Basserdorf, Switzerland), with an energy of 45 kVp, a current of 88 μA and a 200-ms integration time producing a resolution of 20 μm^3 ^voxel size. Each scan comprised a minimum of 500 slices through the entire paraffin block. In order to achieve image noise reduction, a constrained three-dimensional Gaussian filter (sigma 1.2, support 2) was applied. A fixed, global threshold for analysis was chosen that represented the transition in X-ray attenuation between un-mineralized tissue (< 225) and the forming bone (230 - 700). Analysis consisted of defining the outer boundary of the tissue for each 20 mm section in the sample. For consistency, the same settings and thresholds were used for each analysis and applied to every sample in the study. Scout, sagittal and cross-sectional views were examined for evidence of mineralization.

### Histochemical stains

Tissues were fixed in 4% paraformaldehyde, dehydrated, embedded in paraffin and sectioned (6 μm). Paraffin sections were dewaxed, rehydrated and stained with Harris Hemotoxylin (Fisher Scientific, MI, USA; Nos 245-678) and Eosin Y (Fisher Scientific; Nos 245-827) in order to determine cellularity, vascularization and tissue morphology. Sections were also stained with Wright Geimsa (Fisher Scientific; Nos 264-985; phosphate buffer pH 6.8 Nos 262-237) in order to determine the inflammatory cell number and toluidine blue (Sigma, MO, USA; No. 198161) to determine mast cell numbers.

### Immunohistochemistry

Paraffin sections (3 μm) were mounted on Fisher Superfrost/Plus slides which were placed in a 58°C oven for 30 min prior to immunostaining. A Ventana Benchmark XT automated slide stainer (Ventana, AZ, USA; N750-BMKXT-FS) was used for the following immunohistochemical staining reactions. The slide stainer was equipped with an iView DAB detection kit (Ventana) for immunoperoxidase visualization of the targeted antigen. Endogenous biotin reactivity in the tissue sections was blocked using the Endogenous Biotin Blocking kit from Ventana. After the completion of the staining run, the slides were briefly washed in a mild dishwashing detergent solution (Dawn; Proctor & Gable, Ohio, USA) to remove the liquid cover slip solution and processed for haematoxylin counterstaining using a 1:8 dilution of Gills-3 haematoxylin solution (Polysciences, PA, USA) for 1 min. Slides immunostained for 8-OHdG antigen were counterstained with Fast Green Substitute for Light Green Working Solution (Poly Scientific, NY, USA). Specific staining conditions were as follows: p53 mouse monoclonal anti-human antibody (clone: Bp53-11; pre-diluted, 37°C for 32 min) (Ventana; 760-2542) (CC1 treatment* - 1 h); CD68 mouse monoclonal anti-human antibody (clone: KP-1; pre-diluted, 25°C for 32 min) (Ventana; 790-2931) (CC1 treatment* - 30 min); neutrophil elastase mouse monoclonal anti-human antibody (clone: NP57; 1:100 dilution, 25°C for 1 h) (Dakocytomation; M-0752) (no antigen retrieval); 8-OHdG monoclonal antibody (1:20 dilution, 25°C for 1 h) (Oxis International, Inc, CA, USA; 24328) (CC2 treatment* - 8 min then Protease 3 treatment - 16 min); myeloperoxidase mouse anti-human antibody (1:300 dilution, 25°C for 1 h) (Dakocytomation, CA, USA; A-0398) (CC2 treatment* - 1 h); and rabbit anti-nitrotyrosine (1:1000 dilution, 37°C for 1 h) (Harry Ischiropoulos, University of Pennsylvania, USA) (CC2 treatment* - 1 h);. *CC2 is a citrate based buffer, pH 6.0, Protease 3 is a low concentration solution of a serine protease and CC1 is a Tris buffer with 1 mM EDTA (antigen retrieval reagents, Ventana; 950-124). Slides were then mounted in Permount, cover slipped and evaluated by microscopy. Control tissues to determine antibody reactivity and conditions included, human tonsil tissue, bone marrow, normal breast tissue and breast tumour tissue.

### Image acquisition and analysis

For each patient two to three blocks of tissue from each anatomical site were sectioned and complete images of each section (25-30 individual images) were acquired at a magnification of 20×. Images were acquired with a Retiga EXi digital-cooled camera with a red, green and blue electronic filter (QImaging, BC, Canada) or with an RT Color Spot camera (Diagnostic Instruments, MI, USA) on either a Nikon Optiphot or a Nikon E800 (Nikon, NY, USA). Image quantification was performed with Image Pro Plus software (Mediacybernetics, MD, USA), using a customized macro to count diaminobenzidine (DAB) stained cells and nuclei of cells stained with haematoxylin. A quantitative value of the inflammatory response was then presented as the average percent of positive cells (DAB) per total cell number (haematoxylin) normalized to total area. The section results for each block from each anatomical site were averaged and site differences compared.

In order to evaluate the number of macrophages (CD68) and MPO positive cells, the number of each in serial sections was compared. If they existed, the counts were corrected for total cell number and total area of the section.

### Fibroblast isolation

Equal amounts of tissue were taken from each region and combined (total weight ~0.5 g). The 2 × 2 cm pieces were rinsed in Hanks' balanced salt solution to remove any blood, placed in a 25 ml flask and incubated at 37°C for 4 days in α-MEM with 10% fetal bovine serum, 100 units/ml streptomycin and 100 units/ml penicillin (Mediatech, Inc, VA, USA) [66]. Tissue pieces were removed and the media changed on day 4. Cells were passaged when they reached approximately 80% confluency. Cells were not used beyond passage seven.

### RT-PCR and comparative analysis

RNA isolation, quantitative SYBR^® ^Green-based real-time PCR (RT-PCR) oxidative stress and antioxidant defense array and data analysis were performed by SABiosciences (Frederick, MD, USA). Fibroblasts isolated from arthrofibrotic tissue and from primary knee tissue (control) were analysed. The expression level of the three housekeeping genes, β2-microglobulin, β-actin and glyceraldehyde-3-phosphate dehydrogenase, were used to normalize the data presented.

### Statistical analysis

Statistical analysis between groups was performed using a one way ANOVA for normality and student's *t*-test for continuous variables. A level of significance (α), or a *P*-value of less than 0.05, with a 95% confidence interval, was determined. In order to evaluate correlations for patient clinical information and individual test values for each patient, a Pearson correlation coefficient was calculated. For pairs with *P*-values of less than 0.05, there was a significant relationship between the two variables. All parameters were evaluated with SPSS software (version-Base 13.0; SPSS, IL, USA).

## Abbreviations

BMI: body mass index; BV: bone volume; Cl_2_: chlorine gas; CTGF: connective tissue growth factor; HOC1: hypochlorous acid; MGST3: microsomal gluthione S-transferase 3; MPO: myeloperoxidase; μCT: microcomputed tomography; OA: osteoarthritis; PCR: polymerase chain reaction; PNKP: polynucleotide kinase 3'-phosphatase; RNS: reactive nitrogen species; ROM: range of motion; ROS: reactive oxygen species; SOD1: superoxide dismutase 1; TKA: total knee arthroplasty; TrxR1: thioredoxin reductase 1.

## Competing interests

The authors declare that they have no competing interests.

## Authors' contributions

TAF was involved in the study design, analysis and data interpretation and manuscript preparation. JP contributed to the study design, data interpretation and manuscript preparation. CD contributed to the study design, data interpretation and manuscript preparation. MJS was the study coordinator and was involved in the study design, analysis and data interpretation and manuscript preparation.
